# Risk and Outcome after Simultaneous Carotid Surgery and Cardiac Surgery: Single Centre Experience

**DOI:** 10.1155/2018/7205903

**Published:** 2018-08-16

**Authors:** Theodor Tirilomis, Dieter Zenker, Tomislav Stojanovic, Stella Malliarou, Friedrich A. Schoendube

**Affiliations:** ^1^Department for Thoracic, Cardiac, and Vascular Surgery, University of Göttingen, Göttingen, Germany; ^2^Department of Neurology and Neurological Rehabilitation, Asklepios Clinics Schildautal, Seesen, Germany

## Abstract

**Objective:**

Carotid artery stenosis in patients undergoing open-heart surgery may increase risk and deteriorate outcome. The aim of the study was the analysis of risks and outcome after simultaneous carotid and cardiac surgery.

**Methods:**

We retrospectively reviewed the medical records of 100 consecutive patients who underwent simultaneous carotid surgery and open-heart surgery during a 5-year period (from 2006 to 2010). Seventy patients were male and 30 female; the mean age was 70.9±7.9 years (median: 71.8 years). Seventy-three patients underwent coronary bypass grafting (CABG), 18 patients combined CABG and valve procedures, 7 patients CABG combined with other procedures, and 3 patients isolated valve surgery. More than half of patients had had bilateral carotid artery pathology (n=51) including contralateral carotid artery occlusion in 12 cases.

**Results:**

Carotid artery patch plasty was performed in 71 patients and eversion technique in 29. In 75 cases an intraluminal shunt was used. Thirty-day mortality rate was 7% due to cardiac complications (n=5), metabolic disturbance (n=1), and diffuse cerebral embolism (n=1). There were no carotid surgery-related deaths. Postoperatively, transient cerebral ischemia occurred in one patient and stroke with mild permanent neurological deficit (Rankin level 2) in another patient.

**Conclusion:**

Simultaneous carotid artery surgery and open-heart surgery have low risk. The underlying cardiac disease influences outcome.

## 1. Introduction

Stroke after surgery is a feared complication depending on the type and complexity of the procedure [1]. The risk of stroke is increased after cardiac surgery [2, 3]. The cause of brain injury in conjunction with cardiac surgery is multifactorial [1] and carotid artery stenosis is an identified risk factor for postoperative stroke [4, 5]. Therefore, relief of carotid stenosis may decrease postoperative neurologic complications. Over the years, two main strategies developed for the management of concomitant carotid stenosis: the staged and the simultaneous (combined) approach, respectively [6–11]. The aim of the present study was the analysis of the outcome and risk of carotid artery surgery and cardiac surgery combined in our institution. We asked whether this approach is the safe way or whether we expose our patients to an exceptionally high risk.

## 2. Patients and Methods

One hundred consecutive patients (70 male and 30 female) underwent simultaneous open-heart surgery and carotid endarterectomy (CEA) in our institution during 5-year period (2006 to 2010). At the same time, 440 isolated carotid artery surgeries were performed. The clinical data and the outcome were retrospectively reviewed and analysed. The study was performed according to the regulations of the local ethics committee.

The diagnosis of the carotid stenosis was confirmed by Doppler sonography, duplex, and angiography (computed tomography, magnetic resonance, or rarely selective angiography). Cardiac diagnostics included echocardiography and coronary angiography. Indications for cardiac surgery depended on clinical symptoms and the severity of underlying diseases, e.g., unstable or recurrent angina, acute myocardial infarction, dyspnea at rest, or slight exertion.

The criteria for concomitant carotid surgery were as follows:

- in symptomatic patients, stenosis of more than 70%,
- in asymptomatic patients, if stenosis was (a) more than 80% in patients with unilateral stenosis, (b) more than 70% in case of bilateral stenosis or plaque ulcer.

Bilateral stenosis was defined as a contralateral stenosis of more than 50% narrowing.

Carotid surgery was performed before cardiac procedure. At least one of the following techniques of neuromonitoring was applied: transcranial Doppler, somatosensory evoked potentials, or electroencephalogram. An oblique cervical incision was made and the carotid arteries were isolated. After i.v. administration of 5,000 units of heparin, the carotid arteries were clamped. The surgical technique of carotid surgery was according to the decision of the surgeon. In case of patch plasty a longitudinal incision was performed in the common carotid artery and extended to the internal carotid artery beyond the distal extent of the plaque. In case of eversion technique, transection of internal carotid artery was performed at the level of the bifurcation. In both cases, the atherosclerotic plaque was removed in a standard fashion. After carotid artery surgery, cardiac surgery started. In case of coronary artery bypass grafting (CABG) and scheduled use of venous grafts, harvesting of the saphenous vein was performed simultaneously to the carotid surgery. Full anticoagulation (300 units/kg heparin) was achieved before the cardiopulmonary bypass (CPB) was established. In case of valvar and coronary artery surgery, the order of procedure was at the surgeon's discretion. Mostly, the distal venous graft anastomoses were first performed followed by the valvar procedure, then the anastomosis of left thoracic artery graft, and finally the proximal anastomoses. After completing the cardiac surgery, anticoagulation was reversed by protamine sulphate. Then, drainages were placed and the wounds were closed. Postoperatively, patients received heparin (6 to 8 hours after surgery if there is no bleeding) and aspirin from the first postoperative day onwards.

Data are presented as the mean ± standard deviation or median. Early death or early neurologic deficit was defined as an event within 30 days postoperatively. Qualitative data were analysed by using the *χ*^2^ method or the Student's t-test. A probability value p<0.05 was considered to be of statistical significance.

## 3. Results

During the study period 4,791 open-heart surgeries were performed resulting in a 2% occurrence of combined cardiac and carotid artery procedures. Mean age was 70.9±7.9 years (median: 71.8 years). In most cases, severe coronary artery disease was the underlying cardiac pathology (73 patients; Table 1).

**Figure 1:**
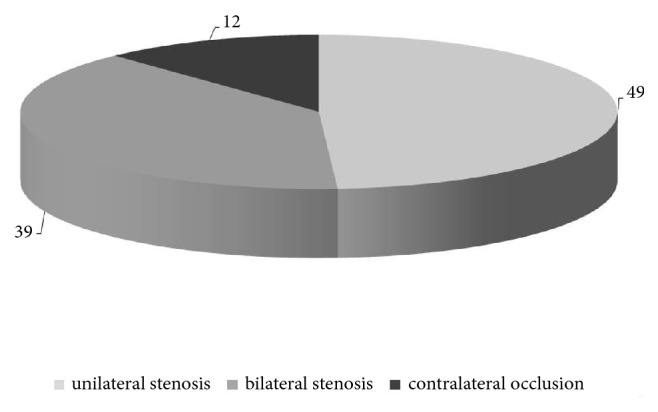
Distribution of carotid stenosis. Most patients suffered from either bilateral stenosis of carotid arteries or stenosis with occlusion of the contralateral artery.

Unilateral carotid stenosis was present in 49 cases, bilateral stenosis was found in 39, and the contralateral carotid artery was occluded in 12 cases (Figure 1). Thirteen patients suffered a stroke preoperatively. We performed CEA with patch plasty (PP) in 71 patients and in eversion technique (ET) in 29 cases. An intraluminal shunt was used in 75 operations (n=64 with PP [90%] and n=11 in ET [40%]).

Seven patients died postoperatively resulting in early mortality of 7% (3 men and 4 women; p=0.104). There were no carotid surgery-related deaths. There was one stroke-related death, however, caused by multiple cerebral emboli of the posterior region. The other causes were of cardiac (n=5) and metabolic (n=1) origins. During surgery, two of these patients needed mechanical circulatory support: each one an intra-aortic balloon pump and an extracorporeal life support system.  Table 2 presents some of the important clinical data of the early deaths. The mean logistic EURO-score of early deaths was 27.6±13.5% (median 25.5%; range from 8.5 to 52.6%). Additionally, two patients suffered from neurological complications (2%). The first patient had a postoperative transient cerebral ischemia but recovered soon after surgery without long-term complications. The second patient had a stroke with permanent neurological deficit (Table 3) but his condition improved after neurological rehabilitation retaining a mild residual deficit (Rankin level 2).

Interestingly, all patients who died suffered from either bilateral carotid stenosis or contralateral occlusion (7 out of 51 cases versus none out of 49 cases with unilateral stenosis; p<0.05).

## 4. Discussion

Stroke after cardiac surgery is one of the most feared complications and its prevention is of the “utmost importance” [12]. The cause of brain injury in conjunction with cardiac surgery is multifactorial [1]. Thromboembolic material, air bubbles, or ruptured calcifications are the main causes of stroke but prolonged intraoperative and postoperative hypotension may increase the risk of stroke as well, due to reduction of cerebral perfusion behind carotid artery stenosis during CPB. However, relief of carotid stenosis decreases neurologic complications after cardiac surgery [13, 14]. The prerequisite for this is, however, that no complication has occurred during carotid surgery. The margin between the benefit and the complication of carotid surgery is narrow. And although the problem of concomitant carotid stenosis in patients undergoing open-heart surgery is well known, the best strategy remains controversial. Current guidelines recommend carotid revascularization in symptomatic patients undergoing coronary artery bypass grafting (CABG) [15]. There is still no consensus for optimal management of patients undergoing other cardiac procedures than CABG and for asymptomatic patients. Only observational but no randomized evidence is available regarding a preferred approach [12]. In December 2010 a multicentre, randomized trial comparing simultaneous carotid surgery and CABG versus isolated CABG in patients with asymptomatic stenosis (CABACS trial) started aiming to recruit 1160 patients with 1:1 block-stratified randomization [16]. Unfortunately, the trial was terminated early after insufficient recruitment (German Clinical Trials Register, ID: DRKS00000521); only 129 patients (intention-to-treat) were enrolled in this study [17]. However, irrespective of small size of the cohort there was no evidence for significant effects for primary and secondary end points in the treatment group [17].

Previous studies reported a mortality and morbidity rate (major cardiovascular adverse events) up to 10 to 12% irrespective of the approach [18]. The staged approach may be performed with carotid surgery followed by coronary bypass grafting (CABG) or the reverse (CABG first and then carotid surgery). However, reported results are still controversial; Coyle et al. [19] reported a high combined stroke and early death rate among patients undergoing simultaneous CEA and CABG while the rate was decreased among patients with the staged approach (26.2% versus 6.6%), while Snider et al. [20] reported low early death and stroke rate of 2% with the simultaneous approach. Yoda et al. [21] reported a different simultaneous approach. They performed the CEA on cardiopulmonary bypass with pulsatile perfusion under moderate hypothermia (nasopharyngeal temperature 32°C) in 15 patients. Their observed neurological complication rate was 6.7%.

Although neurological morbidity may be decreased with the staged approach, patients may be in danger due to cardiac complications (e.g., myocardial infarction) while they are waiting for heart surgery after CEA [22]. Ciangola et al. reported cardiovascular complications in 52% (9 out of 17 patients) before CABG surgery including myocardial infarction in five cases [9]. In a recent study, patients who underwent the staged procedure had a significant higher stroke and myocardial infarction rate than those who underwent cardiac surgery with normal carotid arteries [14].

In our study, bilateral carotid disease was a negative predictor of outcome, probably because bilateral disease is a sign of severity of atherosclerotic disease. Accordingly, all but one of early deaths had severe coronary artery disease.

A problem of simultaneous carotid and cardiac surgery is of course the duration of the whole procedure, since the functional neurological status after the carotid surgery cannot be verified until the patient is awake many hours after the end of the prolonged procedure. In this context, it is important to continue neurological monitoring over the carotid surgery. Near-infrared spectrometry (NIRS) is an interesting novel technique facilitating continuous monitoring even in intensive care units.

An additional important point is regarding the qualification of the operating surgeons. In the present study, the carotid surgeries were always performed by a board-certified vascular surgeon with extensive experience in carotid surgery. In most cases, the operating surgeons were double certified (vascular surgery board and cardiac/cardiothoracic surgery board). The complete surgery (cardiac and carotid procedure) was thus executed by the same surgeon. In the remaining cases, cardiac surgeons continued the procedure after the carotid surgery.

Main limitations of present study are the retrospective design and the small size of the cohort. The number of events after surgery is too small to perform a multivariate analysis.

## 5. Conclusions

For patients suffering from carotid artery stenosis undergoing open-heart surgery, the combined surgical approach is a safe alternative. The underlying cardiac disease determines outcome.

## Figures and Tables

**Table 1:** Cardiac operations performed simultaneously with carotid surgery.

***surgical procedure***	***n=***
isolated CABG *(including Redo-CABG; n= 3)*	73
CABG + AV surgery	15
CABG + AV replacement + Ascending Aorta replacement	1
CABG + AV replacement + LV aneurysm	1
CABG + MV surgery	3
CABG + MV repair + LV aneurysm	1
CABG + Maze procedure	1
Redo-CABG + AV repair + LV aneurysm	1
Redo-CABG + MV replacement + TV repair	1
isolated AV replacement	2
MV replacement + TV repair	1

CABG: *coronary artery bypass grafting*, AV: *aortic valve*, MV: *mitral valve,* TV: *tricuspid valve*, LV: *left ventricle*.

**Table 2:** Clinical data of patients who died after combined surgery within 30 days.

	***sex***	***Age *** *(years)*	***carotid surgery***	***shunt***	***cardiac surgery***	***cause of death***
#1	female	81.4	left - PP	yes	CABG (2x)/ IABP	cardiac
#2	female	70.2	right – PP	yes	CABG (4x)	cerebral embolism (posterior!)
#3	male	68.2	left - PP	yes	Redo: CABG(3x)+AVR+AscAoR/ECLS	cardiac
#4	female	62.9	right – PP	yes	CABG (2x)+AVR+subaortic myectomy	cardiac
#5	male	70.3	left - PP	yes	CABG (3x)	metabolic
#6	female	73.3	right - ET	no	MVR+TV repair	cardiac
#7	male	75.0	left - PP	yes	CABG (4x)+Maze procedure	cardiac

PP: *patch plasty*, ET: *eversion technique*, CABG: *coronary artery bypass grafting*, AVR: *aortic valve replacement*, TV: *tricuspid valve*, AscAoR: *ascending aortic replacement*, IABP: *intra-aortic balloon pump*, ECLS: *extracorporeal life support*.

**Table 3:** Clinical data of patients with neurological complications after combined carotid and cardiac surgery.

***sex***	***Age *** *(years)*	***carotid surgery***	***shunt***	***cardiac surgery***	***symptoms***	***CT scan***	***angiography***
male	72.6	***right*** * – *PP	no	CABG (3x) + AVR	arm paresis	no cerebral ischemia	no obstruction
female	72.9	***left*** * – *PP	yes	CABG (3x)	hemiparesis & aphasia	cerebral infarction	distal occlusion

PP: *patch plasty*, CT: *computed tomography*, CABG: *coronary artery bypass grafting*, AVR: *aortic valve replacement*.

## Data Availability

Row data are available on request. Due to European and national data regulations availability is restricted.
